# Determination of Cellulose Degree of Polymerization in Historical Papers with High Lignin Content

**DOI:** 10.3390/polym13121990

**Published:** 2021-06-17

**Authors:** Jasna Malešič, Ida Kraševec, Irena Kralj Cigić

**Affiliations:** 1 Library Research Department, National and University Library, Turjaška 1, 1000 Ljubljana, Slovenia; 2 Faculty of Chemistry and Chemical Technology, University of Ljubljana, Večna Pot 113, 1001 Ljubljana, Slovenia; ida.krasevec@fkkt.uni-lj.si (I.K.); irena.kralj-cigic@fkkt.uni-lj.si (I.K.C.)

**Keywords:** weight average molecular mass, average degree of polymerization, cellulose, size-exclusion chromatography, viscometry, lignin, chlorite/acetic acid, historical papers

## Abstract

Determination of cellulose degree of polymerization (DP) is one of the most commonly used methods in paper degradation studies, performed either by a standardized method using viscometry (as average degree of polymerization (DP_v_)) or size-exclusion chromatography (SEC) (as weight average molecular mass (M_w_)). Due to the insolubility of papers with high lignin content in cupriethylenediamine (CED), such as groundwood papers, viscometric determination is not possible; therefore, pretreatment is required to allow subsequent dissolution of the papers. In this study, the pretreatment of historical papers containing groundwood with sodium chlorite in acetic acid was investigated, which enables dissolution of the paper samples in CED and determination of the cellulose average degree of polymerization by viscometry (DP_v_). Kappa number was determined to estimate the lignin content in the papers. The suitability of SEC UV-VIS analysis for determination of M_w_ in papers with high lignin content had been verified before it was used as a comparative method for viscometry. Using SEC, changes in the weight average molecular mass (M_w_) of cellulose tricarbanilate (CTC) derivative during delignification were evaluated. The results indicate that no significant depolymerization occurred in the selected samples under the studied delignification conditions, which was additionally confirmed with determination of monosaccharides by ion chromatography. The results of the M_w_ determinations by SEC and DP_v_ by viscometry are in good correlation, justifying the use of viscometry after chlorite/acetic acid pretreatment to determine the cellulose average degree of polymerization in historical papers with high lignin content.

## 1. Introduction

Regardless of the complexity of paper material, the two main chemical degradation pathways are acid hydrolysis and oxidation. Both processes result in cellulose depolymerization, which ultimately leads to a loss of mechanical strength of the paper. The depolymerization of cellulose is primarily followed by determination of the weight average molecular mass (M_w_) or the average degree of polymerization (DP_v_) of cellulose [1]. Determinations are performed either by a standardized procedure using viscometry or size-exclusion chromatography (SEC). Both techniques require sample dissolution, which limits their application. In the pulp and paper industry, conventional viscometry is still the standard approach for the quality control of bleached pulp. It is also quite commonly used by laboratories interested in paper degradation. Indeed, the solution viscosity of pulps allows a simple and easily applicable calculation of the cellulose molecular mass. The most commonly used nonderivatizing cellulose solvents for viscometry include copper(II) complexes with ethylenediamine (CED) or with ammonia (cuoxam) and cadmium ethylenediamine hydroxide (cadoxen) [2].

The three sources of fibrous materials primarily used for historical European paper include groundwood fibers produced directly from wood by thermomechanical processes, rags, or native cotton fibers, and partially delignified cellulose fibers derived from wood [3]. The influence of the lignin content in cellulose pulp on paper durability was recently demonstrated [4]. Contrary to the previous opinions that lignin causes changes in paper due to the hydrolysis of glycoside bonds and oxidation, the authors observed no effects of the lignin content on the degradation rate of paper produced at neutral pH [4]. A different correlation between the decrease in cellulose DP and the loss of mechanical properties was found for contemporary papers compared to the historical groundwood papers subjected to accelerated thermal degradation [5]. The relationship between the DP decrease of cellulose and loss of mechanical properties in the contemporary model groundwood pulp paper sample during accelerated degradation was determined to be linear, whereas an exponential correlation was observed for the historical groundwood pulp paper samples [5]. However, the methods for DP determination of cellulose in papers with high lignin content still represent a challenge for researchers in the field of cultural heritage.

The high lignin content in groundwood pulps makes such papers insoluble in the most common nonderivatizing cellulose solvents. For the native lignin-containing papers for which viscometry is not suitable due to insolubility [3,6,7], i.e., actually for most real archival objects, SEC becomes the only method of choice [1,4,8]; unfortunately, it is not broadly available. SEC of pure cellulose can be carried out after dissolution in suitable solvents, LiCl-DMAC being the most common [9]. It is often necessary to derivatize the cellulosic sample to obtain a soluble material for SEC analysis in a moderately polar solvent, such as tetrahydrofuran (THF) [10]. Hydroxyl groups of the cellulose molecule can be converted to a variety of ethers and esters. These derivatives are often soluble and can theoretically be used for SEC analysis of cellulose derivatives. Thus, it is assumed that the information obtained on the molecular mass distribution of the cellulose derivative accurately reflects that of the original cellulose after appropriate adjustment for increased molecular mass due to a derivative formation. In the past, cellulose trinitrate and to a lesser extent cellulose acetate have been used as derivatives of choice. The tricarbanilate derivative of cellulose is now commonly used for SEC analyses and has several advantages over the nitrate derivative. These advantages include the stability of the tricarbanilate, the complete substitution of the cellulose, the substitution reaction that usually does not cause depolymerization of the original cellulose, and the ready volubility of the carbanilate. Various solvents were investigated as a medium for the reaction of phenylisocyanate with cellulose. No degradation of the cellulose was observed when the reaction was carried out in pyridine at 80 °C [10]. The derivatization with phenylisocyante also has the advantage that the cellulose derivative can be detected by UV detectors, which are more sensitive compared to RI detectors, and it could be employed advantageously when only a small amount of sample is available [11].

Prior to viscometric determination of average DP_v_, attempts were made to partially remove the lignin by chlorite treatment to allow subsequent dissolution of paper made from high-yield pulps [6,12]. It was reported that under the conditions of a modified chlorite method, significant depolymerization of cellulose is prevented, enabling viscometric determination of average DP_v_ in cadoxen solution of cellulose [12]. Viscometry represents a robust, rapid, inexpensive, and efficient technique, but under certain conditions, such as delignification, it can provide data that are subject to systematic error, making it is necessary to compare the results with M_w_ determinations with SEC [13]. Cellulose is a natural macromolecular material; therefore, not all molecules have the same DP, and in complex paper samples where strong macromolecular associations of hemicelluloses and lignin with cellulose are present, an interpretation of the molecular mass distribution rather than an average DP_v_ value is required [14]. Since SEC can provide comprehensive information on the molecular mass distribution, it can be particularly effective in the assessment of the current condition of paper as well as in kinetic studies of cellulose degradation [1].

Different results were obtained when comparing mass average molecular mass determination by viscometry and different SEC methods for cellulose tricarbanilates (CTCs). Some authors describe a good correlation between DP_v_ determination by viscometry and SEC determination of M_w_ of cellulose tricarbanilates [15], while other authors find significant differences between these methods [16].

The degree of polymerization of cellulose is calculated from the intrinsic viscosity of solutions prepared by dissolving cellulose in a suitable solvent, such as CED or cadoxen. In addition to cellulose, paper usually contains other components as well, of which lignin is the most abundant in unbleached wood pulp papers. According to the standard SCAN-CM 15:88 [17], the method for viscometric determination of the degree of polymerization is applicable to all types of pulp that dissolve completely in the CED solution, which is not the case for papers with high lignin content. The applicability of the method is defined in more detail in the Tappi standard [18]. The viscosity of pulp using the capillary viscometer method can be used for conventional pulps with up to 4% lignin. The applicability of this method to pulps with extended delignification has not been determined. It is also noted that chlorite delignification may result in a lower determined viscosity [18]; therefore, the results should be considered approximate only. Pulps with Kappa number above 35 to 40 should be delignified by mild treatment with sodium chlorite or chlorine dioxide to obtain reliable results, as mild treatment minimizes cellulose degradation [18].

Extensive treatment of pure cellulose with the chlorite delignification process may affect the cellulose average DP_v_ [19], which is consistent with a study by Kumar et al. [20]. The two most likely reactions during acid–chlorite delignification are acid hydrolysis of glycosidic bonds and oxidative degradation of polysaccharides [21]; generally, acetic acid is added to the delignification procedure to lower the pH. However, the addition of acetic acid increases the probability of degradation by acid hydrolysis. The presence of lignin in cellulose samples minimized the average DP_v_ reduction of cellulose due to acid-catalyzed cleavage during acid–chlorite delignification [19]. In the study by Mortha et al. [22], acid–chlorite treatment was shown not to induce degradation under room temperature conditions even in bleached Kraft pulps from eucalyptus. In contrast, Kumar et al. [23] demonstrated that delignification of switchgrass and poplar solids under high doses of NaClO_2_ and acetic acid removed significant amounts of carbohydrates and total solids in addition to lignin and that the treatment had a significant effect on cellulose M_w_. The differences in the results of the studies cited above can most likely be explained by different conditions and the different ratios of NaClO_2_, acetic acid, and paper during delignification.

The main components of paper are polysaccharides. Based on quantitative results of monosaccharides in 18th- and 19th-century paper documents determined by HPLC, these papers contained the highest amount of glucose and very small amounts of other hemicellulose components (galactose, mannose, and arabinose and no xylose). The papers from the second half of the 19th to the 21st century contained lower amounts of glucose and up to 16% of other monosaccharides (galactose, mannose, arabinose, and xylose) [24]. Therefore, another parameter to evaluate in the depolymerization of polysaccharides may be the amount of monosaccharides (glucose, mannose, galactose, xylose and arabinose) released during depolymerization. For the determination of small amounts of monosaccharides, ion chromatography is the method of choice [21].

The aim of this study was to evaluate the viscometric method using CED to determine the degree of polymerization in various historical groundwood-containing papers after acid–chlorite delignification. The suitability of SEC UV-VIS analysis for determination of M_w_ of CTC in papers containing high lignin content had been verified before it was used as a comparative method to viscometry. Several paper samples from the books with high content of groundwood fibers were delignified according to the procedure of Kaminska [12]. Lignin removal was followed by the determination of Kappa number, and cellulose degradation was followed by SEC analyses where weight average molecular masses for cellulose tricarbanilate (CTC) derivatives were determined according to the method proposed by Stol et al. [25]. Monosaccharides were determined in filtrates of the different stages of delignification by ion chromatography to additionally evaluate cellulose degradation.

## 2. Materials and Methods

### 2.1. Paper Samples

The paper samples used in the study were purchased from antiquarian bookstores. Fifteen different papers were selected from books published between 1887 and 1987. The publication dates, along with the list of samples, their pH values, and fiber furnish analysis, are presented in Table 1.

### 2.2. Fiber Furnish Analyses

Fiber furnish analyses were performed according to the standard [26] using Nikon Eclipse 80 I (Nikon Instruments Inc., Tokyo, Japan) digital microscope.

### 2.3. Paper Delignification

The delignification of paper was performed according to the literature [12]. The reaction mixture was prepared from 40 mL of distilled water, 0.125 mL of acetic acid (CH_3_COOH, ≥99.8%, puriss p.a.), and 0.375 g of sodium chlorite (NaClO_2_, 80%, puriss p.a.). The paper samples were placed in laboratory glass bottles with screw caps at the ratio of 1 g of paper sample and 40 mL of reaction mixture and thermostated (thermostat VK 2000, Elektromehanika Labonova, Ilirska Bistrica, Slovenia) at 70 °C for 3 or 6 h with constant stirring. After delignification, the reaction was stopped by cooling the suspension in an ice bath. The solutions were filtered through the nylon filter (0.22 μm), and paper samples were washed with distilled water until neutral and dried.

If the dried paper samples did not dissolve in CED after 6 h of delignification, a new portion of the reaction mixture was added to the dried paper sample at the ratio of 1 g of paper and 40 mL of reaction mixture and thermostated at 70 °C for additional 3 h with constant stirring.

### 2.4. pH Measurements

The pH of the water extracts was measured according to the standard of [27], modified to a smaller sample amount [28]: 7 mL of deionized water was added to 100 mg of paper sample. The pH was determined in the water extract after 1 h using a flat membrane electrode (Metrohm 6.0256.100, Metrohm AG, Herisau, Switterland) connected to a Mettler Toledo MP 220 (Mettler Toledo, Columbus, OH, USA) pH meter.

### 2.5. Kappa Number

Kappa number, related to the lignin content, was determined according to the TAPPI 236 om-13 standard [29] adapted to a smaller sample amount (0.10 ± 0.02 g).

### 2.6. Viscometry

Viscometric determinations of the average degree of polymerization (average DP_v_) were performed according to the standard viscometric methods [17,30], using a glass capillary viscosimeter (Schott and Gen, Mainz, Germany) and fresh CED solvent (Carlo Erba Reagents S.r.l., Milan, Italy). Twenty milligrams of paper was dissolved in 20 mL of solution, consisting of 10 mL cupriethylenediamine (CED) and 10 mL deionized water. The efflux time of a solution was measured in duplicates. The Mark–Houwink–Sakurada equation was used to calculate average DP_v_ [31].

### 2.7. Determination of Weight Average Molecular Mass

The molecular mass of cellulose tricarbanilates (CTCs) [25,32] was determined using size-exclusion chromatography. For the preparation of cellulose tricarbanilate, about 3 mg of the material was removed using a drive punch. The sample was dried at 105 °C for 2 h, after which 1 mL of pyridine and 0.1 mL of phenyl isocyanate were added. After 48 h at 80 °C, the reaction was stopped by adding 0.1 mL of methanol. Before injection into the chromatographic system, the reaction mixtures were filtered with 0.45 μm Nylon syringe filters and diluted with tetrahydrofuran (THF) to 0.27 g·L^−1^ (with respect to cellulose). Typical RSD of duplicate determinations was 5%.

The size-exclusion chromatography (SEC) analyses were performed on an 1100 Series HPLC system 1100 (Agilent, Santa Clara, CA, USA) equipped with a degasser, a binary pump, an auto-sampler, and a UV-VIS detector set at 235 nm for the determination of cellulose carbanilates and at 210 nm for the determination of polystyrene standards. The separation was achieved on two Jordi Gel DVB mixed bed columns with dimensions 250 mm × 10 mm, preceded by a 50 mm × 10 mm Jordi Gel DVB precolumn (Jordi Labs, Mansfield, MA, USA) at 35 °C with tetrahydrofuran (THF) as the mobile phase at a flow rate of 1 mL/min. The injection volume was 50 μL. Chromatographic data were processed with Cirrus software (Marlow, United Kingdom). Polystyrene standards (PSs) (PSS Polymer Standards Service GmbH, Mainz, Germany)) were used for preparation of standards solutions containing 0.1 g·L^−1^ of the individual standard in THF. The first standard solution contained PSs with the following molecular masses (M_w_): 564,000, 64,000, and 8210 g·mol^−1^, the second standard solution contained 2,530,000, 226,000, and 34,000 g·mol^−1^ and the third contained 1,070,000, 125,000, and 17,500 g·mol^−1^. All chromatographic results are expressed as weight average molecular mass of CTC (M_w_) relative to polystyrene standards.

### 2.8. Determination of Monosaccharides

Monosaccharide determination was carried out in the first filtrates after 3 or 6 h of delignification of paper samples. After 6 h of delignification, the paper sample was flushed and then dried, and a new portion of reaction mixture was added to the paper sample. After an additional 3 h of delignification (9 h altogether), monosaccharide determination was performed in the second filtrate. All filtrates were additionally filtered through 0.45 μm filters (Chrom4 GmbH, Suhl, Germany) before injection into an ion chromatograph, Dionex ICS-5000 (Thermo Fisher Scientific, Waltham, Massachusetts, United States), consisting of a gradient pump and an electrochemical detector Dionex ED (Thermo Fisher Scientific, Waltham, MA, USA) with gold working electrode and Ag/AgCl reference electrode. A Dionex CarboPac PA20 3 mm × 150 mm analytical column (Thermo Fisher Scientific, Waltham, MA, USA) was used, with the mobile phase composed of MQ (A), 10 mM NaOH (B), and 100 mM NaOH in MQ (C); 0.5 mL/min flow; and 25 μL sample injection volume. Separation was performed at isocratic conditions at 20% B and 80% A for 20 min, followed by a wash step (increase to 100% C in 3 min, hold for 30 min, decrease to 20% B and 80% A in 3 min) and finally re-equilibration to starting conditions for 14 min. Each sample was injected in duplicate, and standard quadruple carbohydrate pulsed amperometry waveform was used for detection (*E*_1_ = +0.1 V from 0 to 0.4 ms, *E*_2_ = −2.0 V from 0.41 to 0.42 ms, *E*_3_ = +0.6 V from 0.42 to 0.43 ms, *E*_4_ = −0.1 V from 0.44 to 0.5 ms). The monosaccharides were quantitatively determined in filtrates using calibration in the range 0.1–1 mg·L^−1^ (glucose, xylose, and arabinose), 0.5–1.1 mg·L^−1^ (galactose), or 3–6 mg·L^−1^ (mannose). Limit of detection (LOD) was determined to be 0.03 mg/L for glucose, xylose, and arabinose; 0.17 mg·L^−1^ for galactose; and 1 mg·L^−1^ for mannose.

## 3. Results and Discussion

The examined real paper samples are presented in Table 1. The papers were taken from the books published between 1887 and 1983, produced in an acidic environment as indicated by the pH values presented in Table 1. Fiber furnish analysis revealed different compositions of groundwood and sulfite pulp. Three paper samples (No. 5, No. 6, and No. 15) also contain cotton fibers (below 28.5%).

### 3.1. Determination of Kappa Number and Solubility in CED

The Kappa number [29] is an important parameter for determining the residual lignin content in a paper made from finished or in-process pulp. It is thus a parameter indicative of the completeness of pulping process for many types of chemical and semichemical pulps, both bleached and semibleached. The Kappa number is based on the reaction of a highly oxidizing chemical, potassium permanganate, with lignin as well as a low content of certain other organic impurities that remain in the pulp at various stages of its processing. The Kappa number provides valuable information to both the manufacturer of the pulp and the papermaking user of the pulp about the properties of the pulp as well as the paper made from it, especially the content of residual lignin present [33].

The Kappa number of the pulp was determined for all samples before and after delignification (Table 2) according to the procedure of Kaminska [12]. The standard applies to many types of chemical, semichemical, unbleached, and semibleached pulps within the Kappa number range from 1 to 100. Above a Kappa number of 100, the accuracy of the test, as well as the relationship between Kappa number and lignin content, may decrease. Therefore, for two paper samples (No. 1 and No. 5), where the determined Kappa values were above 100, the exact values are not given. In Table 2, it is only indicated that these two samples exceed the value of 100.

Kappa numbers were the highest in the samples containing the highest percentage of groundwood (samples No. 1 and No. 5) and the lowest in the sample (No. 13) with the lowest content of groundwood pulp (Table 1). The Kappa number decreases with delignification time for all the samples studied, as evident from Table 2. Solubility in CED was tested before and after delignification of the samples for 6 h (and for sample No. 1 also after 3 h). In the cases where the delignified samples did not dissolve in CED, a fresh portion of the reaction mixture was added, and the time of delignification was prolonged for an additional 3 h. In the study by Kumar et al. [23], during delignification of different cellulose biomass samples with higher concentration of reagents (0.6 g sodium chlorite and 0.6 mL acetic acid per gram of paper; in our case, the ratio was 0.375 g sodium chlorite and 0.1254 mL acetic acid per gram of paper) and fresh loading of reagents added every 2 h, more than 90% of the lignin was removed after 6 h. In the study, reaction time beyond 6 h appeared to have low impact on further delignification [23]. The differences in delignification of investigated samples can be attributed to different concentrations of the reagent used in our study.

As evident from Table 2, all samples with lower Kappa numbers, i.e., less than 23, completely dissolved into CED. According to the standard SCAN-CM 15:88 [17], the method for viscometric determination of the average degree of polymerization can be used for all types of pulps that dissolve in the CED solution.

According to the Tappi standard [29], for pulps below 70% total pulp yield, the Klason lignin content (in %) is approximately equal to the Kappa number ×0.13. If the Kappa number is to be used to determine a precise numerical value for the lignin content in a particular pulp, more specific relationships have to be developed for each pulp type, since the relationship varies with wood type and delignification procedure [33,34]. Since the relationship cited above [29] has not been proven and specific relationships have not been determined for each pulp used in the present study, we can only roughly estimate [33] from the results presented in Table 2 that the lignin content that might still be present in the paper samples should be lower than 3%.

### 3.2. Stability of Cellulose during Delignification

**Figure 1:**
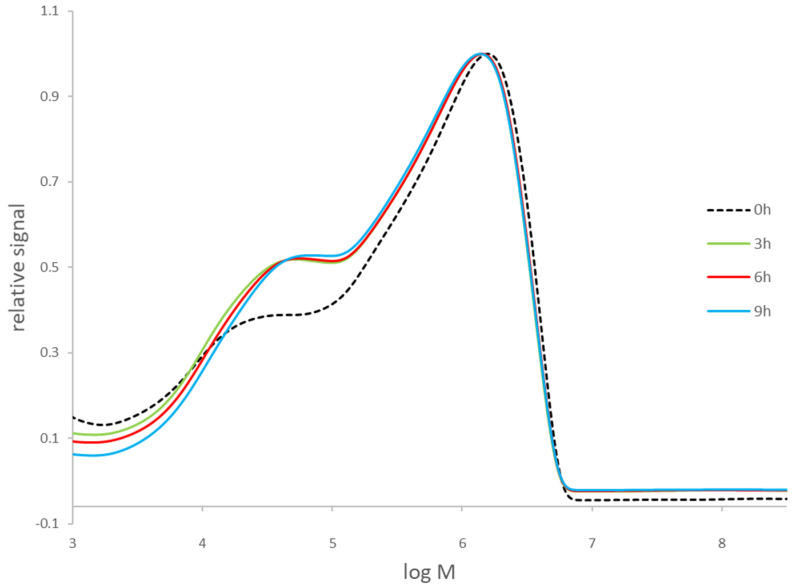
Molecular mass distribution of CTC derivatives for sample No. 1, untreated (0 h) and after delignification for 3, 6, or 9 h.

The degradation of paper samples during the process of delignification was followed by SEC using a UV-VIS detector. Figure 1 shows the relative signals of the CTC derivatives for the untreated and treated paper sample No. 1 for 3, 6, and 9 h. The CTC fraction appears as the highest molecular mass, and its distribution is much wider than that of the fraction of hemicellulose tricarbanilates, which have lower molecular masses [11]. The SEC analysis of Whatman filter paper No. 1 confirmed that the signal with broader distribution represents molecules of cellulose CTC derivatives (data not shown). From Figure 1, it can be concluded that the changes in molecular mass of cellulose after delignification for 3, 6, or 9 h are not significant (for sample No. 1, the M_w_ difference is 1%).

Kappa number of sample No. 1 was reduced from over 100 to 19 during delignification (Table 2). From the CTC signal of the untreated sample No. 1, which is only slightly different from the signals of the delignified samples, it can be concluded that the molecular mass of cellulose is not significantly changed, but differences in the lower molecular mass range are observed, especially between the untreated and delignified paper samples (Figure 1, Sample No. 1, untreated and after delignification for 3 h). The hemicellulose distribution is slightly reduced with longer delignification (Figure 1, delignification for 3, 6, or 9 h), suggesting that the delignification procedure could influence the degradation of hemicelluloses.

Therefore, to evaluate the degradation of the cellulosic and hemicellulose polymer due to chlorite oxidation, the content of monosaccharides in the undiluted filtrates of the reaction mixtures after delignification was determined by ion chromatography for paper sample No. 1 (Table 3). The monosaccharides detected were glucose, galactose, xylose, and arabinose, whose amounts in the first filtrates after 6 h of delignification were higher than after 3 h of delignification, as expected. Although their amount in both first filtrates is very low, the results are in agreement with the results of SEC (Figure 1). In the second filtrate, the amounts of monosaccharides were even lower, which is to be expected since a new portion of the reaction mixture was added. The content of monosaccharides released during delignification (Table 3) is also low compared to the average values of monosaccharides previously determined in different paper documents [24]. The content of glucose released after 6 h of delignification is less than 0.00005%, which is not significant compared to over 70% of glucose content measured in papers produced from 1950 to 2007 [24]. The content of arabinose after 6 h of delignification is less than 0.005%, which is also low compared to average amounts of arabinose in papers produced during the same period (1.54–1.7%) [24]. Therefore, the results presented in Table 3 additionally confirm that the degradation of cellulose or hemicelluloses during acid/chlorite treatment is not intense under the described conditions.

The results are consistent with those reported in the literature [19], where samples of Kraft linerboard pulp exposed to the acid–chlorite delignification procedure were analyzed to evaluate degradation during treatment. The Klason lignin content of the unbleached linerboard pulp was 19.1%. The pulp samples were exposed to the delignification process several times. The molecular mass distribution curves showed that after two delignification treatment cycles there was no degradation of the cellulose, resulting in pulp with 3.2% Klason lignin content. After further delignification treatment cycles, the signal of the cellulose shifted to lower molecular masses, indicating significant cellulose degradation. The distribution in the hemicellulose range narrowed after each delignification cycle, indicating a significant decrease in hemicellulose content in the pulp [19]. In this study, the chromatogram of the untreated sample is not shown. The differences in the curves between untreated and treated paper samples can be explained by the fact that none of the methods, neither derivatization in the form of cellulose tricarbanilates nor SEC of cellulose samples in LiCl-DMAc, seem to be directly suitable for the case of impure cellulosic samples, such as mechanical pulps or paper with a high content of hemicelluloses and lignin [9]. Therefore, also before SEC analysis, delignification of cellulosic samples was attempted using chlorite-based pretreatments either at 70 °C and pH 4.8 (holocellulose treatment) or using cold holocellulose treatment [9]. It was shown that the holocellulose treatment resulted in a significant decrease in cellulose average DP, but the presence of lignin counteracted this effect [9].

A high lignin content could interfere with the SEC measurements made with a UV-VIS detector, since lignin absorbs in the UV-VIS region due to its aromatic nature [1,4]. However, in Figure 1, which shows the SEC chromatograms of the paper sample No. 1 with the highest lignin content of the samples studied (Table 1) at different stages of delignification, almost no difference can be observed between the relative signals in the molecular mass range of cellulose. Therefore, it can be concluded that lignin content does not interfere with the determination of cellulose M_w_ after CTC derivatization and analysis using our SEC setup. SEC with UV-VIS and MALS detectors have already been used for DP determinations of cellulose in lignin-containing paper samples [4]. Analyses of CTC derivatives have also been performed for papers with much higher lignin contents (up to 30%) using SEC with MALS and UV-VIS detector [35].

For all paper samples, the average molecular mass of CTC derivatives was determined using SEC before and after delignification to evaluate cellulose degradation due to delignification (Table 4). The differences between the determined weight average molecular masses of the papers before and after delignification were less than 10%. In addition to the above explanation of the differences in SEC chromatograms of untreated and treated cellulose samples, previous studies have shown that when derivatized tricarbanilate cellulose samples are analyzed in the same laboratory, the average RSD between measurements of the same sample is 8%, but RSD between laboratories is higher with a value of 15% [11]. The differences in the obtained molecular masses can also be attributed to the uncertainty due to the preparation of the cellulose tricarbanilate derivatives, which is the common sample preparation for cellulose dissolution before SEC analysis. A precondition for derivatization prior to SEC analyses is that the chemical reaction proceeds with quantitative yields, which is suggested for the CTC derivative and pure cellulose. Derivatization is not straightforward if the lignin content is high; a low content does not interfere. The lignin content could be a contributory factor responsible for the overestimated M_w_ values in the cellulose tricarbanilate derivatives in the conventional calibration, but it cannot be the only or the decisive one. However, the results showed that derivatization of pure cellulose produced the most homogeneous derivative (RSD 1%) and the untreated dissolving pulp caused less problems compared to its bleached version (RSD 4.5 vs. 12%) [11].

The differences in weight average molecular masses (Table 4) are not significant and can also be attributed to the inhomogeneity of the paper samples; thus, for the selected samples, it can be concluded that delignification does not induce any significant cellulose degradation. It has been demonstrated that degradation is more pronounced for new papers than for aged or historical paper samples, as only minor differences in average DP_v_ determination occurred in aged newsprint during 5 h of delignification [12]. In the study by Kumar et al. [20], it was reported that the acid–chlorite delignification procedure caused a drastic reduction in the average degree of polymerization for cellulose from filter paper. The same author demonstrated that sodium chlorite delignification of various cellulose biomass samples under a higher concentration of the agents reduces the molecular mass of cellulose [23]. Other studies [9,19] have proved that in a conventional lignocellulosic sample, lignin protects the polysaccharides from degradation by acid hydrolysis and oxidative cleavage during acid–chlorite delignification. However, once the lignin content is reduced to below about 1%, the polysaccharides are susceptible to degradation. At this point, the acid–chlorite treatment begins to affect the hemicelluloses and eventually attacks the cellulose component; at levels below 1% lignin, delignification can significantly damage the polysaccharide components [19]. It can be concluded that for the selected lignocellulosic historical paper samples, where the lignin content was reduced to a value of about 3%, no significant depolymerization of cellulose was observed during acid–chlorite delignification for up to 9 h, in agreement with the literature data [19].

### 3.3. Viscometric Determination of Cellulose Average DP_v_ of Delignified Paper Samples

**Figure 2:**
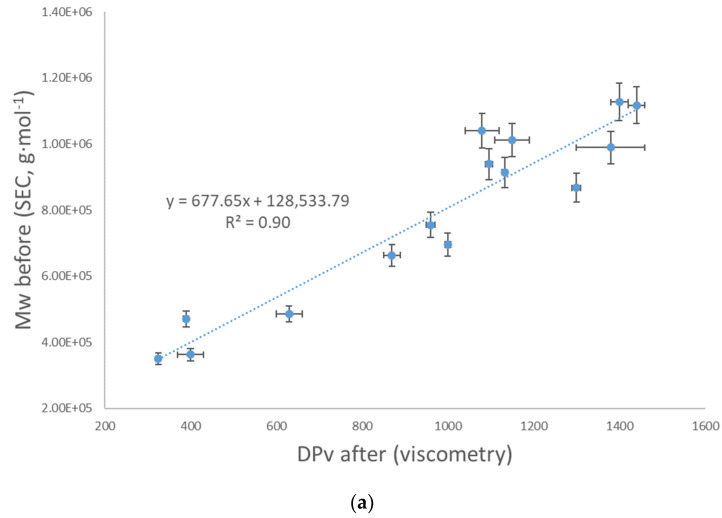

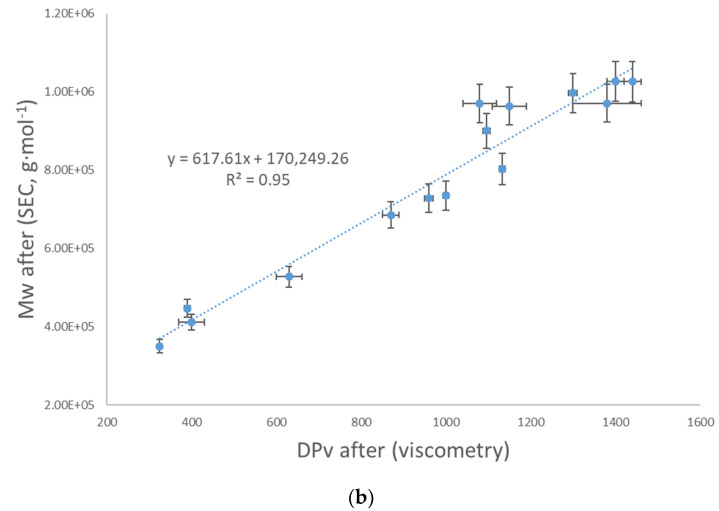
Mass average molecular mass of CTC derivatives of cellulose determined by SEC before (**a**) and after (**b**) delignification vs. average DP_v_ of cellulose determined using viscometry after delignification of paper samples described in Table 1. Error bars represent standard deviations of duplicate determinations.

After delignification of paper samples and their complete dissolution in CED, the average DP_v_ was determined by viscometry. The results correlate with both molecular masses of CTC derivatives before and after delignification (Figure 2a,b), which is consistent with the small differences in weight average molecular masses before and after delignification (Table 4). Therefore, the average DP_v_ of cellulose in lignin-containing paper samples can be reliably determined by viscometry.

### 3.4. Reliability of the Results

Both values, M_w_ determined by SEC with UV-VIS detector and average DP_v_ determined by viscometry, are susceptible to various systematic errors due to sample preparation, detection, and interpretation of the results; therefore, the results can only be considered as relative and not absolute values.

As previously described, derivatization involves a substitution of free hydroxyl groups of cellulose with phenyl isocyanates to form cellulose tricarbanilates (CTCs) for analysis in tetrahydrofuran. Generally, it is believed that these methods do not cause degradation of cellulose; however, during dissolution or derivatization, some structural changes and loss of some fractions may occur, which in turn lead to inaccurate determination of the actual molecular mass of cellulose in the sample examined [36]. Derivatization is also not straightforward when the lignin content is high; low content does not interfere. A high lignin content may be partly responsible for the overestimated M_w_ values of cellulose tricarbanilate derivatives in conventional calibration [11].

Several authors have compared different methods for determining the molecular mass of cellulose. Dupont and Mortha [16] studied pure cellulose (Whatman paper No. 1) in cadoxen during accelerated degradation by viscometry and by SEC using different detectors: MALS, differential refractive index (RI) for LiCl-DMAc solutions, and low-angle light scattering (LALS) and UV for the CTC derivatives. The highest values for the DP were obtained with the SEC of LiCl-DMAc solutions, lower values were obtained with the SEC of CTC derivatives, and the lowest values were obtained with viscometry. A good correlation between viscometry in CED and SEC of CTC derivatives with MALS detection for pure cellulose as well as for softwood bleached cellulose was found by Łojewski et al. [8]. In the study by Kačík et al. [36], three methods for the analysis of carbanilated cellulose derivatives dissolved in THF were compared: SEC with MALS detection, SEC in combination with diode-array detector and calibration with polystyrene standards (SEC DAD), and asymmetric flow field–flow fractionation in combination with a multiangle light scattering (A4F-MALS). Each of the methods provided different absolute values of molecular mass, but the mutual correlations between them were linear with high correlation coefficients (r = 0.990–0.992); thus, each of the methods studied can be useful for M_w_ or DP determinations in monitoring changes in cellulose in lignocellulosic materials. A comparison was made on the basis of results obtained with different detectors (MALS and/or UV-VIS) in analyses of various paper samples [37]; it included paper, a representative of typical lignin-containing acidic paper used in books of the 19th and 20th centuries. A high lignin content in paper samples that absorbs in the UV-VIS region [1] could interfere with M_w_ determinations of cellulose using SEC UV-VIS, making the approach unsuitable for the analysis of lignin-containing samples [11]. However, prior to SEC with UV-VIS detector, the CTC solutions are filtered with syringe filters, which remove most of the lignin [37]. The softwood paper samples show a bimodal distribution because the SEC UV-VIS system detects hemicelluloses with lower molecular mass than cellulose, while the MALS UV-VIS detector neglects the hemicellulose fraction. The results obtained with SEC UV-VIS were found to be reproducible, as demonstrated by the interlaboratory comparison between our laboratory and authors’ laboratory [37]. The maximum relative error between the results for a large number of historical paper samples was 30% in the lower mass range, while the minimum error was 10% for the upper mass range. Using different data processing methods and SEC, it was again proved that molecular masses should be treated only relatively [37]. For direct dissolution of nonderivatized cellulose and CTC, a round-robin test of cellulose SEC was performed using RI, MALS, and viscometry detectors [11]. The obtained weight average molecular mass values showed a variation of 36% across all methods. The two most important influencing parameters were sample preparation (derivatization and dissolution methods) and the type of molecular mass evaluation in SEC, i.e., by calibration or absolute measurements using light scattering. Both dissolution approaches gave acceptable results when the correct procedures were used [11].

In our research, the average molecular masses of carbanilated cellulose were determined from SEC analysis by calibration with polystyrene standards. The molecular masses are, therefore, relative values, since the SEC separation is based on the hydrodynamic volume of the analytes, which is an approximation of the molecular mass. Potthast et al. [11] have shown that overestimation of molecular mass is mainly due to differences in hydrodynamic radii in solutions of CTC derivatives as compared to calibration samples, such as pullulan or polystyrene. In previous studies, the weight average molecular masses (M_w_) were compared with those determined using SEC coupled to a multiangle light scattering photometer (SEC MALS), and a fairly good correlation between the results was observed [38]. In addition to the systematic errors obtained with the UV-VIS detector, it is important to consider that although intralaboratory repeatability (precision) is high, the interlaboratory reproducibility (accuracy) is not as good [11,37].

Gaussian curve deconvolution in the range representative for cellulose, hemicelluloses, or aggregates [23] was not applied to the molecular mass distribution curves because, as described in the previous section, cellulose signals do not change significantly during delignification. According to the results of SEC, the hemicellulose contents in the paper samples were significantly lower, less than 30%. Moreover, in paper samples with highly degraded cellulose, only the signals of hemicelluloses and low molecular masses of cellulose derivatives were observed after SEC analysis, which cannot be properly deconvoluted. It was therefore decided not to recalculate the weight average molecular masses to avoid additional systematic errors due to the deconvolution of the molecular mass distribution curves. Although the degradation of hemicelluloses during delignification could systematically lower the Mw values of cellulose obtained without deconvolution, it is assumed that the systematic error is negligible based on the results of SEC and the determination of monosaccharides in the undiluted filtrates (Table 3).

The average DP_v_ values determined using viscometry are systematically lower, which is probably the consequence of noncellulosic components in the paper samples. They contribute to the mass of the sample but not to the viscosity of the analyte. Additional systematic errors can also be attributed to the degradation of oxidized cellulose samples in the CED solvent [39,40], although some researchers claim that, contrary to these findings, viscometry gives results that are generally consistent with those obtained using SEC [1], even for the most degraded samples.

## 4. Conclusions

In the present study, the use of viscometry to determine the degree of polymerization of groundwood-containing pulps was evaluated. A significant number of samples were examined; however, the same trend was observed for all samples studied. It was demonstrated that paper samples can be dissolved in cupriethylenediamine (CED) when lignin content calculated from the Kappa number is lower than 3%; consequently, the average DP_v_ of cellulose can be determined with viscometry. The degradation of cellulose by delignification is more pronounced in the absence of lignin; therefore, complete removal of lignin is not required.

As viscometry may provide data subject to systematic errors under certain conditions, such as delignification, is it necessary to compare the results of average DP_v_ determinations with those obtained using SEC. The results of the viscometric determinations of average DP_v_ are in good correlation with the M_w_ determined by SEC both before and after delignification.

The process of delignification of paper samples was followed using SEC with UV-VIS detector. From the SEC chromatograms of the paper samples with lignin content reduced from 15 to 2.4%, it was evident that the lignin content did not interfere with the determinations of cellulose M_w_ after the preparation and analysis of cellulose tricarbanilates (CTCs) using our SEC setup. Under delignification conditions as presented in the study (0.375 g sodium chlorite and 0.125 mL acetic acid per gram of paper), no significant depolymerization of cellulose was observed in the paper samples which is confirmed by the results of the SEC analyses and monosaccharide determination in the filtrates of the reaction mixtures.

The study suggests that the method of delignification of paper samples by Kamniska [12] can be used prior to viscometric determination of average DP_v_ in CED in paper degradation studies of historical papers containing groundwood, when relative results would be sufficient. Therefore, a robust, inexpensive, and efficient classical method of determination of average DP_v_ by viscometry can be applied to the historical groundwood-containing samples, and the instrumental technique SEC is not required.

With the investigated set of historical paper samples and the limitations associated with their accessibility for additional analysis, it is planned to expand the existing historical sample set and conduct further research to obtain absolute results.

## Figures and Tables

**Table 1:** List of real paper samples, publication years, pH values, and fiber furnish analysis. The pH values are calculated from two measurements with an average standard deviation of 0.1 pH units.

Sample No.	Year of Publication	pH Value	Groundwood Fiber Content (%)	Bleached Chemical Pulp (%)	Cotton Fibers (%)
1	1983	5.1	85	15	
2	1960	6.4	66	34	
3	1958	6.2	65	35	
4	1903	4.8	64	36	
5	1982	5.3	84	14	2
6	1987	5.6	45	26.6	28.5
7	1953	6.9	58	42	
8	1965	5.8	64	36	
9	1889	4.9	81	19	
10	1899	5.1	61	39	
11	1898	5.3	58	42	
12	1896	5.3	68	32	
13	1909	5.5	35	65	
14	1911	4.9	66	34	
15	1887	4.6	57	20.5	22.5

**Table 2:** Kappa number of paper samples before and after 3, 6, or 9 h of delignification and their solubility in CED (+ indicating completely soluble in CED). The Kappa number is calculated from three measurements with an average RSD of 4%.

Sample No.	Time of Delignification (h)	Kappa Number	Solubility in CED
1	0	above 100	
	3	44	
	6	38	
	9	19	+
2	0	97	
	6	19	+
3	0	97	
	6	18	+
4	0	72	
	6	8	+
5	0	above 100	
	6	42	
	9	18	+
6	0	75	
	6	16	+
7	0	76	
	6	20	+
8	0	92	
	6	32	
	9	11	+
9	0	81	
	6	25	
	9	7	+
10	0	62	
	6	10	+
11	0	57	
	6	16	+
12	0	81	
	6	20	+
13	0	19	+
	6	3	+
14	0	24	
	6	3	+
15	0	82	
	6	23	+

**Table 3:** Monosaccharide content in the first filtrates after 3 or 6 h of delignification and second filtrates after additional 3 h of delignification (9 h altogether).

Monosaccharide	First Filtrate, 3 h	First Filtrate, 6 h	Second Filtrate, 9 h
glucose	0.11 mg·L^−1^	0.12 mg·L^−1^	<LOD
mannose	nd	nd	nd
galactose	nd	<LOD	nd
xylose	nd	<LOD	nd
arabinose	<LOD	1.16 mg·L^−1^	nd

nd = not detected.

**Table 4:** Weight average molecular mass (M_w_) for CTC derivatives of the samples before and after delignification. The measurements were performed in duplicates. Relative standard deviation for M_w_ determinations was below 5%.

Sample No.	M_w_ Before Delignification (mg·L^−1^)	M_w_ After Delignification (mg·L^−1^)	M_w_ Difference (%)
1	989,500	971,000	1
2	1,117,000	1,026,000	6
3	1,127,000	1,027,000	7
4	486,000	527,500	6
5	867,000	997,000	10
6	1,011,500	963,000	3
7	939,000	900,500	3
8	914,500	803,000	9
9	755,000	727,500	3
10	663,000	685,500	2
11	695,500	735,000	4
12	1,040,500	970,000	5
13	471,000	447,000	4
14	351,000	350,500	below 1
15	363,000	412,000	9

## Data Availability

The data presented in this study are available on request from the corresponding author. The data are not publicly available because sharing and archiving research data in a publicly accessible repository has not been established yet.
